# 

**Published:** 1877-02

**Authors:** 


### CONTENTS:

ARTICLE I.—The Amalgam Question. By Prof. Hodgkin.................... 433

ARTICLE II.—The American Academy of Dental Surgery.................... 438

Facial Neuralgia. By Dr. C. P. Hunt.................... 439

ARTICLE III—Alveolar Abscess. By G. D. Pollock, F R. C. S.................... 443

ARTICLE IV.—New York Odontological Society.................... 456

ARTICLE V.—Chloroform and Dentistry.................... 464

ARTICLE VI.—To the Dental Profession.................... 467

### EDITORIAL, ETC.

The Dentists' Future.................... 470

Alumni Notice.................... 471

Bonwill's Method of Anæsthesia.................... 472

Obliteration of Deformity from Depressed Cicatrices.................... 472

### BIBLIOGRAPHICAL.

The Scientific American.................... 473

The Students' Guide to Dental Anatomy and Surgery.................... 474

The American Agriculturist.................... 475

The Physicians' Visiting List for 1877.................... 475

Micro-Photographs in Histology.................... 476

The Sun, N. Y., for 1877.................... 476

Vick's Floral Guide for 1877.................... 477

The Pen and Plow.................... 477

### MONTHLY SUMMARY.

Valuable Mouth Washes.................... 478

Repairing Thermometers.................... 478

Tooth Paste.................... 478

The Toxic Properties of Glycerine.................... 479

The Function of the Uvula.................... 479

Influence of Anæsthetics upon the Sexual Impressions of Females.................... 479

Is Alcohol Food.................... 480

Cremation as a Science.................... 480

